# Thymol as an Intestinal Antiseptic

**Published:** 1890-10

**Authors:** 


### SELECTIONS.

### Thymol as an Intestinal Antiseptic.

The *Medical News*, in an editorial on "Intestinal Antiseptics," speaks with special approval of thymol, naphthol and salicylate of bismuth. It says: "There is none better than the first mentioned, which is perfectly innocuous in large doses and possesses an antiseptic power four times greater than that of carbolic acid. Its value as an antiseptic seems to have been more fully recognized in Italy than elsewhere, and has been endorsed by Martini, Bufalini, Testi and others. In our opinion it is, when properly administered and in suitable doses, *facile princeps* among intestinal antiseptics. Salicylate of bismuth is also an admirable remedy of this sort, and is the one most approved by Dujardin-Beaumetz in the article mentioned. He usually administers it in capsules in combination with magnesia, sodium bicarbonate, prepared chalk, phosphate of lime, beta-naphthol, or charcoal."—*Boston Med. and Surg. Journal.*

